# Pulmonary transplant complications: a radiologic review

**DOI:** 10.1186/s13019-024-02731-w

**Published:** 2024-05-03

**Authors:** Samuel Friedlander, Brian Pogatchnik, Yuka Furuya, Tadashi Allen

**Affiliations:** 1 Department of Radiology, University of Minnesota Medical School, Minneapolis, MN 55455 USA; 2 Department of Radiology, Stanford University School of Medicine, Stanford, CA 94305 USA; 3 Medical Director of Lung Transplant, CareDX, Inc, Brisbane, CA 94005 USA

**Keywords:** Complications, Pulmonary, Transplant, Radiology, Cardiothoracic

## Abstract

Lung transplantation has become the definitive treatment for end stage respiratory disease. Numbers and survival rates have increased over the past decade, with transplant recipients living longer and with greater comorbidities, resulting in greater complexity of care. Common and uncommon complications that occur in the immediate, early, intermediate, and late periods can have significant impact on the course of the transplant. Fortunately, advancements in surgery, medical care, and imaging as well as other diagnostics work to prevent, identify, and manage complications that would otherwise have a negative impact on survivability. This review will focus on contextualizing complications both categorically and chronologically, with highlights of specific imaging and clinical features in order to inform both radiologists and clinicians involved in post-transplant care.

**Supplementary Information:**

The online version contains supplementary material available at 10.1186/s13019-024-02731-w.

## Introduction

Following the first human lung transplant performed by Dr. Hardy and his team at the University of Mississippi in 1963, the number of lung transplants has increased steadily, particularly over the last few decades [1–3]. From 2010–2018, approximately 34,000 were reported worldwide. Over half of those were done in North America alone [3–5]. Efforts to increase available donors have contributed to the rising rates of transplants. Worldwide, chronic obstructive pulmonary disease (COPD) is the most common indication for lung transplant, however in North America idiopathic pulmonary fibrosis (IPF) has supplanted COPD, followed by cystic fibrosis (CF) and alpha-1 antitrypsin deficiency (A1ATD) [3, 6].

In the early 2010s, the median, 1-, and 5-year survival rates had all increased from previous decades in part due to advancements such as infection prophylaxis, immunosuppression, and surgical techniques reducing incidence of morbid airway complications [7–10]. However, early complications remain dominated by infection and acute rejection, while chronic lung allograft dysfunction (CLAD), infection, and malignancy significantly affect late survival [7, 11]. Contributing to these complications are an increase in recipients of advanced age and low volume centers performing transplants [3, 7, 12]. Further, survival is lower than that of other solid organ transplant recipients [13].

Lung transplant recipients now live longer and are burdened with a greater number of comorbidities than in prior decades, lending to a greater volume and complexity of postoperative imaging. As imaging can support and potentially diagnose these complications, review of time-course specific features is of value to radiologists and transplant clinicians involved in post-transplant care [2, 14].

### Overview of complications

**Fig. 1:**
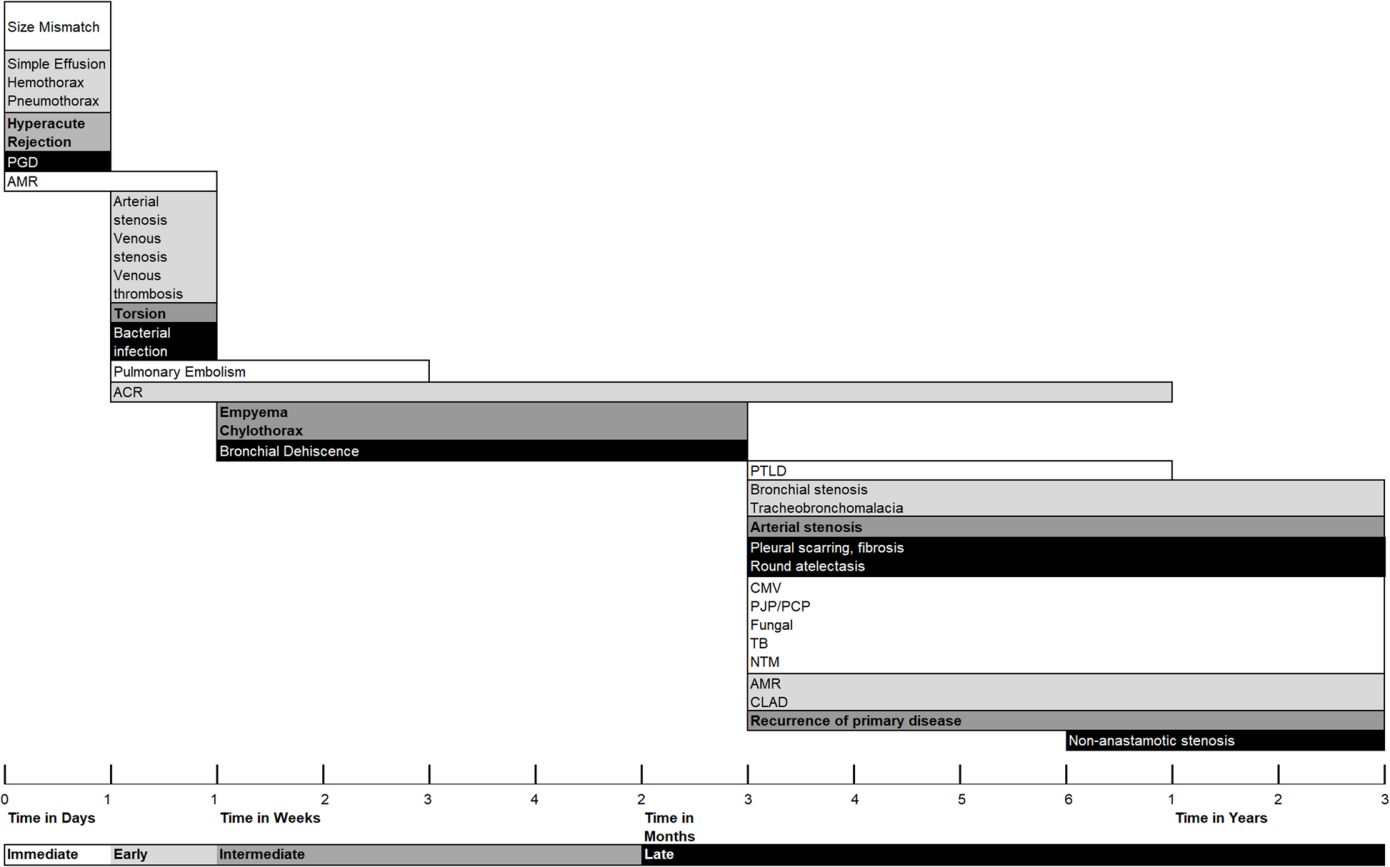
Posttransplant Complications Over Time. Note.–- ACR = acute cellular rejection, AMR = antibody mediated rejection, CLAD = chronic lung allograft dysfunction, CMV = cytomegalovirus, PGD = primary graft dysfunction, PJP/PCP = pneumocystis jirovecii pneumonia/pneumocystis pneumonia, PTLD = posttransplant lymphoproliferative disorder, NTM = nontuberculous mycobacteria, TB = tuberculosis

Complications can be categorized based on time course (as well as specific imaging features or mechanism of pathology (Table 1, Fig. 1) [15]. An approximate timeline serves this purpose: immediate, early, intermediate, and late [15].

**Table 1:** Timing, Categorization, and Incidence of Posttransplant complications

Complication	Timing after surgery	Incidence
**Mechanical**		
Size mismatch	Preoperative-perioperative	60% of single lung transplants 50% of bilateral transplants^a^
Torsion	Days	Rare reports
**Airway**		
Bronchial dehiscence	Weeks-Months	1–10%
Bronchial anastomotic stenosis	Months	1.6–32%
Non-anastomotic stenosis	Months	2.5–3%
TBM	Months	1–4%
**Vascular**		
Pulmonary embolism	Days-Months	1–19.5%, up to 27% on autopsy
Arterial stenosis	Days-Months	< 2%
Venous stenosis	Days	1.4%
Venous thrombosis	Days	2.5%
**PGD**		
	Hours-Days	30%
**Pleural**		
Simple effusion	Hours-Days	
Pneumothorax	Hours-Days	
Hemothorax	Hours-Days	
Chylothorax	Weeks	< 1–11%
Empyema	Weeks-Months	
Scarring Fibrosis Round atelectasis	Months	
**Infection**		
Bacterial infection	Days	
B. Cepacia	Pre and perioperative	3–6% cystic fibrosis patients
CMV PJP/PCP Fungal TB, NTM	Months	
**Immunologic**		
Hyperacute rejection	Intraoperative-Hours postop	Rare reports
ACR	Days-Weeks	27.3%
AMR	Hours-Days	47%
CLAD	Months-Years	50% at 5 years and 76% at 10 years
**Recurrence**		
Sarcoidosis (most common)	Months-Years	1/3 of sarcoidosis 1% overall
**Malignancy**		
PTLD	Months-Years	3–9%
Primary lung cancer	Months-Years	1–9%

*ACR* Acute cellular rejection, *AMR* Antibody mediated rejection, *B*. *Cepacia* Burkholderia Cepacia, *CLAD* Chronic lung allograft dysfunction, *CMV* Cytomegalovirus, *PJP/PCP* Pneumocystis jirovecii pneumonia/pneumocystis pneumonia, *PTLD* Posttransplant lymphoproliferative disorder, *NTM* Nontuberculous mycobacteria, *TB* Tuberculosis, *TBM* Tracheobronchomalacia

^a^In patients with chronic obstructive pulmonary disease and alpha 1 antitrypsin deficiency

Radiographs are ordered for preoperative baseline imaging and subsequently for evaluating support device positioning, complications such as effusions, edema, and infection, and after bronchoscopy [2, 16]. Radiographs are widely and immediately available in a range of clinical settings, deliver minimal radiation, and are highly cost effective. Computed tomography (CT) may be preferred for problem solving and specific clinical scenarios [2, 17]. Transplant specific imaging protocols after discharge are variable though routine radiography is suggested by some to screen for late complications [16, 17]. Therefore, radiographic assessment of complications are presented herein and highlighted, where possible, in the text and figures.

### Mechanical

Size mismatch between donor allograft and recipient thoracic cavity can be apparent during surgery as well as in the immediate postoperative period, though prevention of significant size mismatch occurs during preoperative planning [2]. Size difference of up to 25% is acceptable for bilateral lung transplants (BLT); for single lung transplants (SLT), this is not well defined [18]. Rates of mismatch vary with indication for transplant, as the size of the thoracic cavity diverge between obstructive and restrictive lung disease. The 36th International Society for Heart and Lung Transplantation (ISHLT) report detailed that 60% of SLTs and 50% of BLTs for recipients with COPD/A1ATD came from oversized donors [6].

Recipients of oversized lungs can present with persistent hypoxemia and a requirement for prolonged mechanical ventilation that may be mitigated by graft volume reduction prior to implantation [19]. Oversized donor lungs can result in atelectasis and scarring once transplanted, while undersized donors can result in hyperinflation or persistent pleural complications such as pneumothorax and pleural effusion [2, 9].

Pulmonary torsion is reported primarily in case reports in the early postoperative period [2, 20]. Patients can present with acute cardiopulmonary instability consisting of increased pulmonary artery pressure (PAP), hypoxemia, and hypotension [21, 22]. Risk factors include an undersized allograft resulting in a large space for the lung to torse, complete fissures with separate lobes, and challenging cases requiring longer vascular conduits with greater manipulation and anatomic complexity [2, 20]. Radiography reveals a change in lobar positioning with atelectasis/consolidation and hilar displacement over subsequent exams [2, 21]. Contrast enhanced CT or CT angiography (CTA) better depicts these findings in addition to swirling, abnormal positioning, or cutoff of the vascular pedicle and bronchi [2, 14, 15, 20]. Due to its infrequency it has the potential to be misdiagnosed and lead to infarction and rejection if surgical correction is not performed within 12 hours [2, 14, 15, 20].

### Airway

The 2018 ISHLT consensus on airway complications reported an incidence of 2–18% based on recent studies [23]. Mortality is 2–4% [24]. The pathophysiology involves decreased systemic blood supply and relative ischemia in the immediate postoperative period [9, 15]. Anastomotic blood flow is dependent on low retrograde flow from the pulmonary arterial supply at the time of surgery, as bronchial arterial supply is not often surgically re-established [25]. Bronchial neovascularization takes up to 6 weeks [23, 25].

Anastomotic dehiscence most commonly occurs within the first month after surgery [2, 15]. Incidence is 1–10%, which has decreased from prior decades [23, 25]. Despite this, many centers still report high morbidity and mortality associated with severe cases of dehiscence [23, 24, 26–28].

Risk factors that promote ischemia include infection, prolonged ventilation, primary graft dysfunction (PGD), use of certain immunosuppressants (mechanistic target-of-rapamycin (mTOR) inhibitors), and surgical technique – a long bronchial donor stump, short recipient, and telescoping anastomosis [14, 29].

**Fig. 2:**
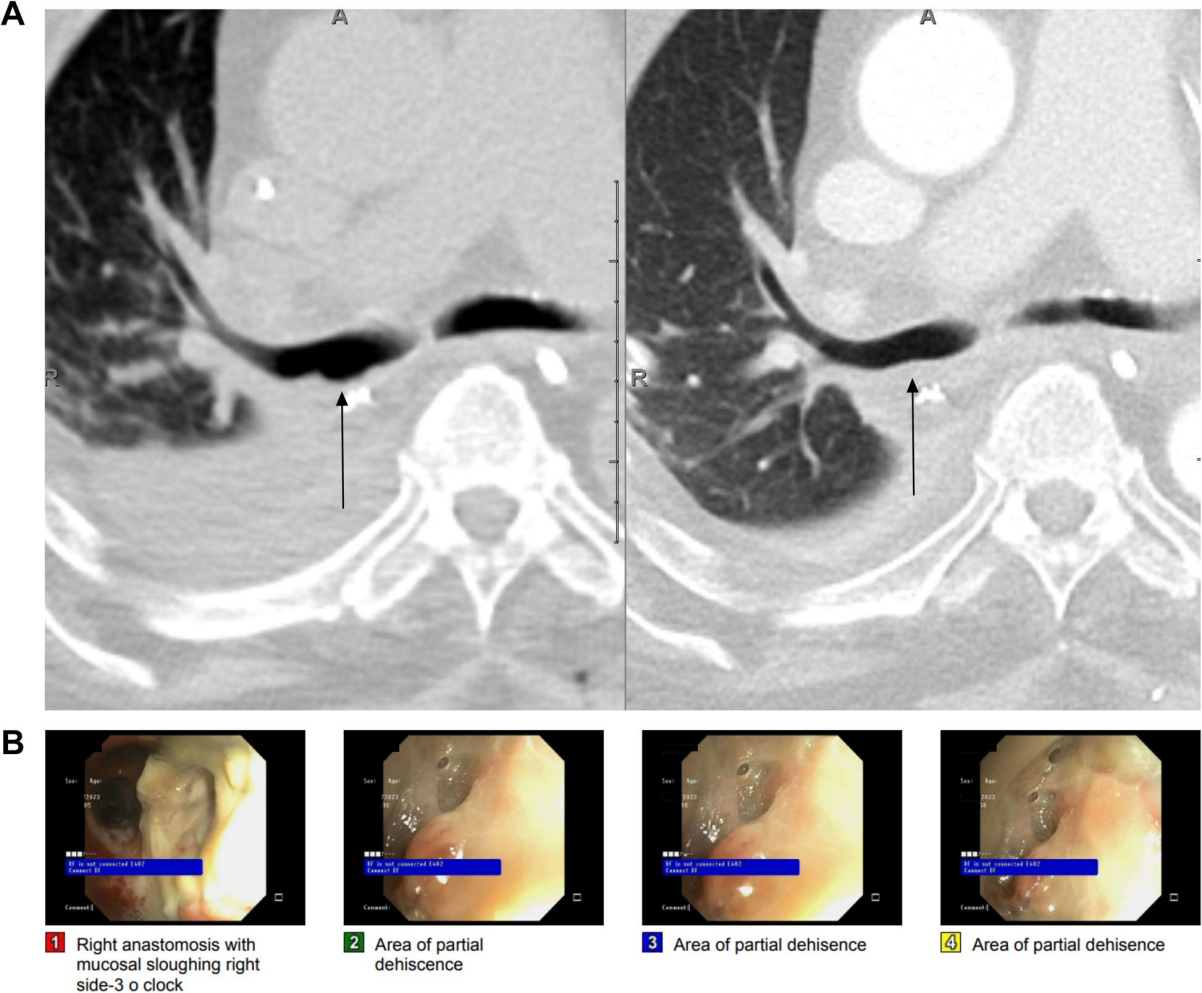
Partial bronchial anastomotic dehiscence in a 71-year-old male. **A** Comparison axial CT images (lung windows) show an abnormal contour of the posterior right bronchial anastomosis and membranous portion of the perianastomotic region (left, black arrow) 2 weeks after bilateral lung transplantation, which was new from the previous scan obtained 1 week after transplant with a normal appearing anastomosis (right, black arrow). There was no extraluminal air or pneumomediastinum. **B** Bronchoscopy performed just prior to the left image in (**A**) revealed membranous dehiscence which was 0–25% circumferential and ischemia/necrosis which was 51–100% circumferential, prompting the CT

Dehiscence can present as persistent air leaks, subcutaneous emphysema, or need for prolonged mechanical ventilation [29]. Radiographs usually suggest dehiscence indirectly by the persistent or delayed appearance of extraluminal air [2, 29]. Persistent atelectasis and infection are also consequences of dehiscence [29]. CT depicts the anastomotic defect with bronchial wall irregularity and extraluminal peribronchial air, pneumomediastinum, and pneumothorax [29, 30]. A focal anastomotic contour abnormality alone is also possible (Fig. 2). Flexible or rigid bronchoscopy is performed for diagnosis, and, in some cases, intervention [2, 14, 29].

**Fig. 3.**
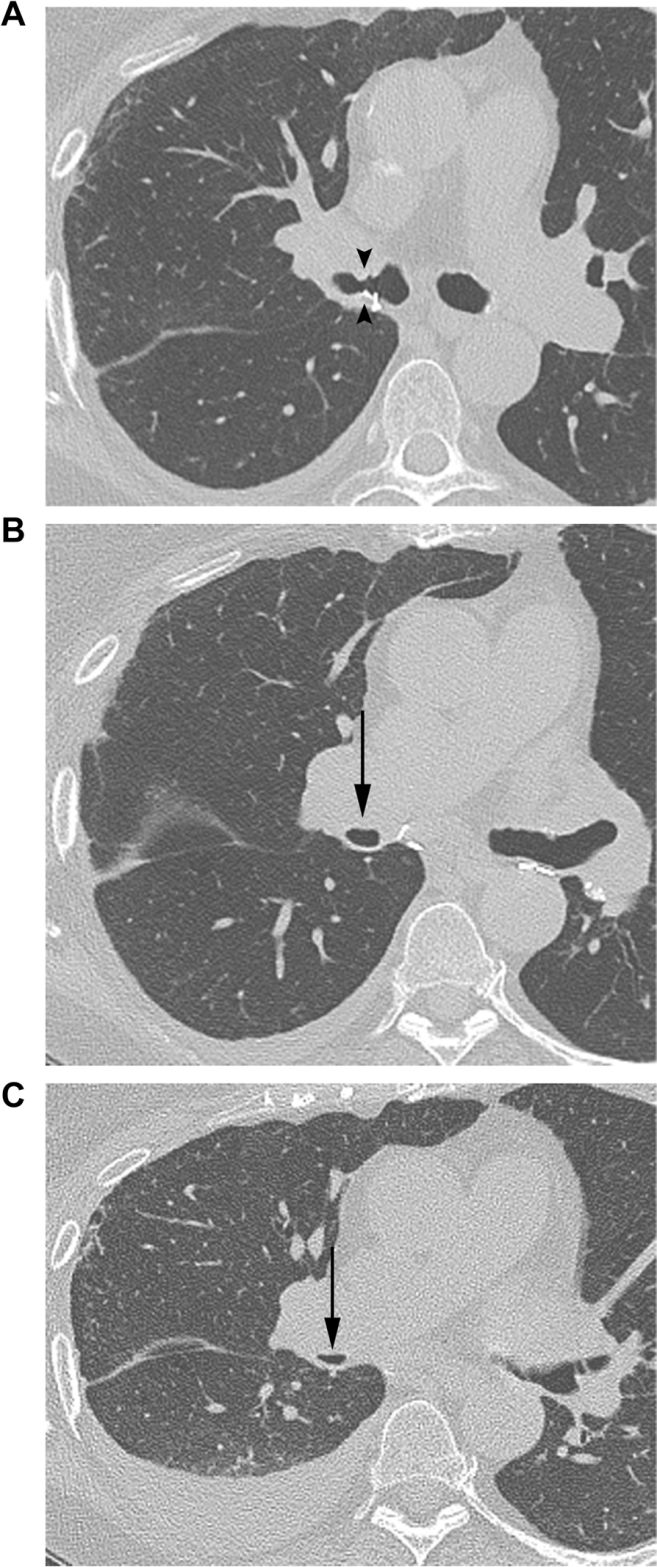
67-year-old male who underwent bilateral lung transplants for COPD with bronchial stenosis and bronchomalacia. **A** Axial CT image (lung window) shows right sided bronchial anastomotic stenosis (arrowheads), previously discovered on bronchoscopy. **B**,** C** Inspiratory (**B**) and expiratory (**C**) axial CT images in lung window show a patent bronchus intermedius (arrow in **B**) with collapse of the anterior cartilaginous portion on expiratory phase (arrow in **C**) suggesting bronchomalacia

Bronchial stenosis is the most common airway complication, and usually presents after 2–3 months [2, 14]. Incidence ranges from 1.6–32%, with a wide range due to heterogeneous reporting prior to the 2018 ISHLT consensus [23–25, 29]. Central airway stenosis affects the region within 2 cm of the anastomosis, with rarer reports of distal/non-anastomotic stenosis beyond 2 cm [23]. Associations include healed dehiscence, infection, telescoping anastomosis, and repetitive trauma from bronchoscopy [15, 29]. Patients may present with wheezing, asymptomatic airflow obstruction on spirometry, productive cough, and in cases of complete occlusion, post-obstructive pneumonia [29, 30]. Radiographs depict post obstructive lobar atelectasis and pneumonia [31]. CT will demonstrate fixed anastomotic narrowing (Fig. 3) [15, 29]. > 50% reduction in cross-sectional area is considered significant [23].

**Fig. 4:**
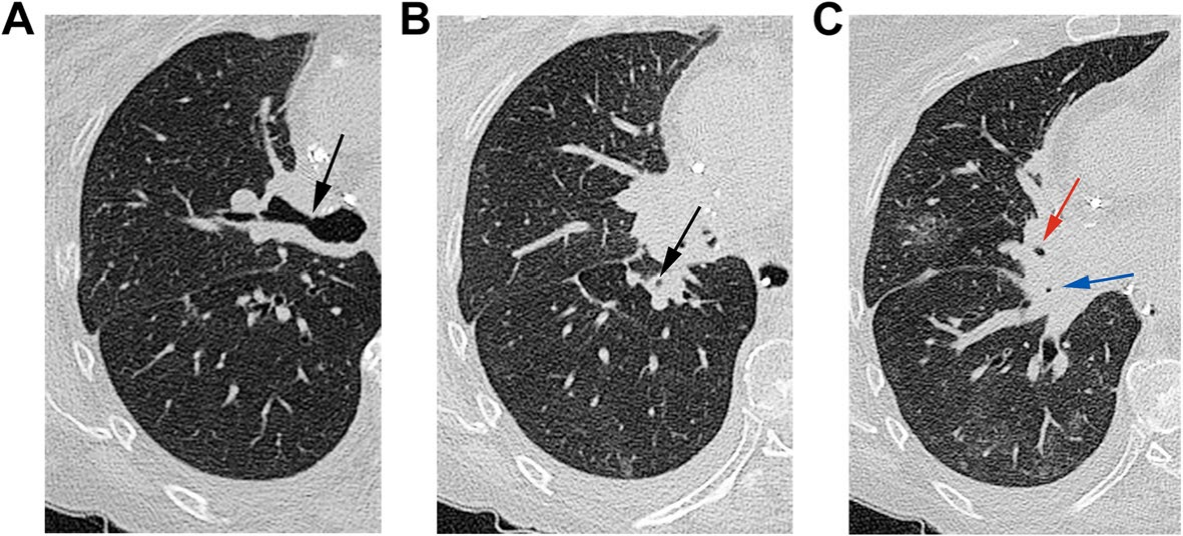
Multiple non-anastomotic bronchial stenoses in a 61-year-old female who underwent bilateral lung transplants for alpha-1 antitrypsin deficiency. **A** Axial CT image (lung window) shows narrowing of the proximal right upper lobe bronchus (arrow). **B** Axial CT image (lung window) shows narrowing of the bronchus intermedius (arrow). **C** Axial CT image (lung window) shows narrowing of the right middle (red arrow) and lower lobe (blue arrow) bronchi. Stents were subsequently placed in the right lobar bronchi, as well as the left mainstem and lobar bronchi (stenoses in these not shown)

Distal/non-anastomotic stenosis is reported in 2.5–3% of transplants [29]. The most commonly described form is vanishing bronchus intermedius syndrome (VBIS) and occurs approximately 6 months after transplant [29]. Risk factors include ischemia and infection, though the exact cause is not known [23]. VBIS presents with persistent fever, pneumonia, and airflow obstruction, with imaging demonstrating post-obstructive atelectasis or air trapping (Fig. 4) [14]. > 50% luminal area narrowing is significant [23], with the bronchus intermedius most commonly involved secondary to its narrow lumen [14]. Although imaging can aid in diagnosis of airway stenoses, bronchoscopy is confirmatory and allows for intervention [29].

Tracheobronchomalacia (TBM) has an incidence of 1–4% and occurs within 4 months after transplant [24, 29]. Pathologically it is defined as > 50% luminal area reduction with expiration, within 1 cm of the anastomosis (peri-anastomotic), or beyond (diffuse) [25]. Some have reported TBM’s association with airway stenosis, and diffuse TBM with bronchiolitis obliterans syndrome (BOS) [29]. Risk factors include prolonged intubation, inflammation, recurrent infections, and trauma [32]. The exact mechanism is unknown but involves the loss of cartilaginous airway support [2, 29].

TBM can present with dyspnea, productive cough, and variable airflow obstruction on spirometry [29]. Imaging can aid in diagnosis, and CT protocols that include dynamic exhalation and virtual bronchoscopy reconstructions improve visualization of airway collapse and prevent underestimation of luminal narrowing and length of involvement (Supplementary Tables 1 and 2) [10, 32–34]. The primary finding is airway collapse with expiration, with an additional qualitative feature of collapse of the anterolateral cartilaginous rings (Fig. 3) [32]. An incidentally discovered lunate shaped trachea on routine/inspiratory CT should raise suspicion for TBM [34]. The severity of collapse and presence of concomitant infection or rejection may influence treatment [25]. Bronchoscopy with visualization of airway collapse during expiration remains the gold standard for diagnosis [29].

### Vascular

Vascular anastomotic complications are reported in 2–15% of transplants [35, 36]. Pulmonary embolism (PE) and arterial stenosis are the most common [36]. Clinical features consist of unexplained hypoxemia or prolonged need for mechanical ventilation, dyspnea, pulmonary hypertension, and hemodynamic compromise [36]. A high index of suspicion is needed, especially for rare complications such as vascular stenosis, as signs and symptoms can be indistinguishable from other early post-transplant complications such as infection or PGD [36].

PE is most common in the early and intermediate postoperative time periods. In lung transplant recipients there is higher incidence, morbidity, and mortality from venous thromboembolism (VTE), including deep venous thrombosis and PE, thought to be a result of poor or absent collateralization of bronchial circulation [2, 30, 35, 36]. Reported post-transplant incidence is 1–19.5% compared to 1.6% in the general surgical population [36–38]. Risk factors include older age, diabetes, recent pneumonia, use of immunosuppressants such as sirolimus, and prolonged mechanical ventilation [39, 40].

**Fig. 5:**
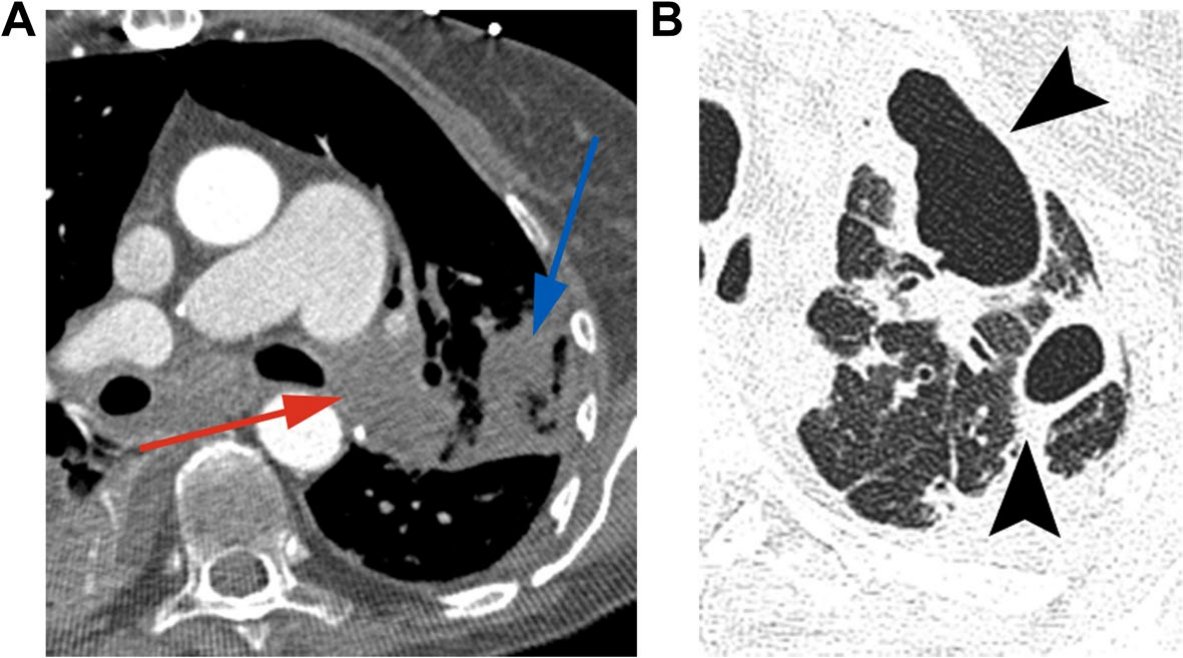
Pulmonary embolism and infarct in a 59-year-old female one year out from lung transplants for fibrotic hypersensitivity pneumonitis. **A** Axial contrast-enhanced CT image timed for the pulmonary arteries shows a large filling defect occluding the left pulmonary artery (red arrow). An associated subpleural consolidation represents pulmonary infarction (blue arrow). **B** Followup axial CT image (lung window) shows evolution of pulmonary infarct to cavitations most pronounced in the left lung apex (arrowheads)

Symptoms include tachycardia, dyspnea, cough, and hemoptysis [30]. Radiographic findings are neither specific nor sensitive. On CT pulmonary angiogram, a filling defect within or occlusion of the pulmonary arteries is diagnostic. Secondary features include oligemia, mosaic attenuation, and/or wedge-shaped subpleural consolidative or ground glass opacities representing infarcts (Fig. 5) [30, 41].

In retrospective studies, PE was independently associated with greater in-hospital death compared to those without VTE [42], and within the first 180 days, strongly associated with bronchial stenosis and CLAD [43]. Complications include infarction and graft loss [2, 30, 36]. The risk of pulmonary infarction is greater than in the general population due to delayed bronchial revascularization, with incidence up to 37.5% [38, 44]. Infarction may be associated with cavitation (Fig. 5), with resultant empyema, abscess, bronchopleural fistula, and pneumothorax [44, 45].

Arterial anastomotic stenosis can be seen both early and late after transplant and occurs in less than 2% of cases [36]. Risk factors include a short allograft artery, long vascular pedicle, suturing technique, or thrombus, in combination with natural tortuosity of the pulmonary arteries [15, 36]. A systematic review found an estimated mortality rate of 22.6% [46].

**Fig. 6:**
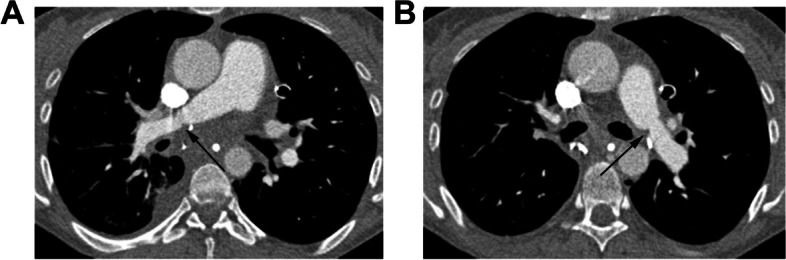
29-year-old female who underwent bilateral lung transplants for cystic fibrosis who developed pulmonary hypertension and pulmonary artery stenosis 3 weeks after surgery. **A-B** Axial contrast-enhanced CT images timed for the pulmonary arteries (**A, B**) show stenoses in the right (black arrow in **A**) and left (black arrow in **B**) pulmonary arterial anastomoses. Right heart catheterization showed elevated pulmonary artery pressures proximal to the stenoses with widened mean pressure gradients across the stenoses consistent with pulmonary hypertension. Stents were subsequently placed (not shown)

Clinical presentation includes hypoxemia, pulmonary artery hypertension (PAH), and hemodynamic compromise including hypotension [46]. CT and magnetic resonance (MR) angiography show focal narrowing at the anastomosis (Fig. 6), and nuclear medicine ventilation perfusion imaging shows a perfusion defect with intact ventilation, or ventilation/perfusion mismatch [15]. 75% anastomotic diameter narrowing is hemodynamically significant. Interstitial edema and pleural effusions are commonly associated [36].

Pulmonary vein thrombosis is an early complication [15, 36]. A recent systematic review reported incidence and mortality of 2.5% and 24% respectively among a total of 1,618 transplants from 34 studies. Risk factors are not well established [47]. A filling defect on delayed venous phase chest CT is diagnostic. Secondary imaging findings include persistent parenchymal opacities and pulmonary edema [15]. Complications include infarct, allograft failure, and stroke via systemic embolization [48].

Venous anastomotic stenosis occurs early, within days [15, 30]. Stenosis is currently without standardized criteria for diagnosis. A case report identified 18 cases from the literature [49], while a systematic review of the literature reported a prevalence of 1.4%, with an estimated mortality rate of 45% [47]. Pulmonary venous thrombosis is a risk factor [36]. Radiographs show airspace opacification confined to the affected lobe. Delayed venous phase CT demonstrates focal anastomotic diameter narrowing, greater than the expected 1–2 mm folds, most commonly at the left inferior pulmonary vein [36]. Secondary signs include consolidations, ground glass opacities, and interlobular septal thickening representing interstitial edema [15, 30, 36].

Patients with PAH receiving transplants can expect normalization of pulmonary artery (PA) dilation over a period of months, which correlates with normalized PAPs [50]. Right ventricular (RV) dysfunction should immediately improve, while characteristics of severe dysfunction that can be noted on imaging such as RV dilation, RV hypertrophy, and pericardial effusion should decrease or resolve over months [51]. Conundrums include a higher prevalence of PGD and edema in the early setting and PA dilation due to size mismatch, PAH recurrence, or the aforementioned vascular complications such as anastomotic stenosis [36, 52]. CTA and CT venography are adjuncts to echocardiography and right heart catheterization and can evaluate patency and size of the pulmonary vasculature, the anastomoses, and lung parenchyma [32].

### Primary graft dysfunction

PGD can occur in the early to intermediate postoperative period, usually radiographically evident at 24–72 h with resolution by 5–10 days [2]. PGD requires exclusion of other causes of acute respiratory distress syndrome (ARDS) [2, 11]. The 2005 ISHLT consensus statement and subsequent 2016 revision defined PGD based on PaO2 to FiO2 ratio, radiologic findings of pulmonary edema, timing of presentation, and exclusion of other pathologies including infection, hyperacute rejection, and cardiogenic pulmonary edema [11].

Estimated incidence is 30% [53]. A single center retrospective review which looked at incidence and outcome of grade 3 PGD utilizing the 2016 criteria showed a 90-day mortality of 8.5% and a 1-year mortality of 25.5% [54, 55]. PGD is associated with many risk factors, among which include a prolonged ischemic time of the donor allograft and hemodynamic instability after brain death [53, 56]. A preoperative diagnosis of PAH is also a significant risk factor [55, 57].

**Fig. 7:**
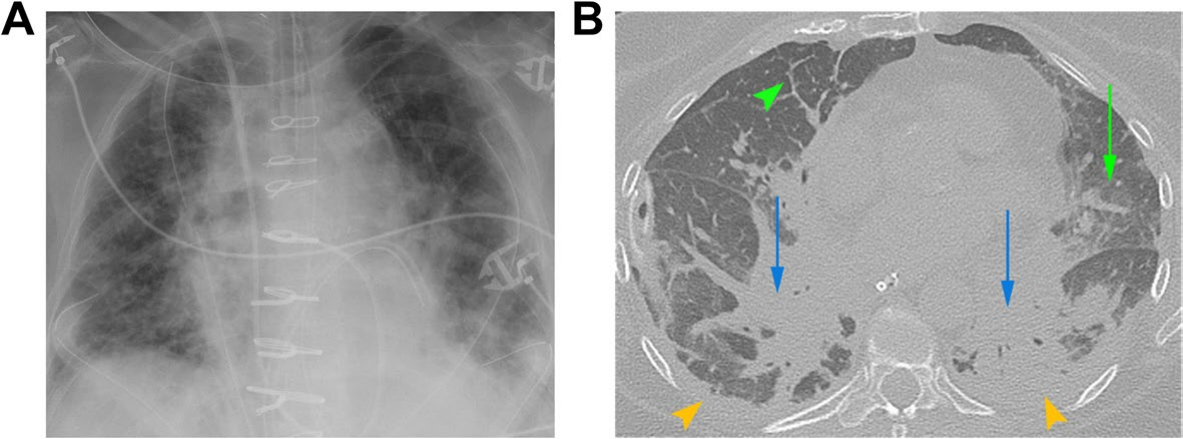
PGD in a 62-year-old female with bilateral lung transplants. **A** Frontal radiograph obtained on postoperative day 2 after worsening respiratory status including respiratory alkalosis and increased FiO2 requirements shows bilateral hazy opacities and interstitial markings consistent with pulmonary edema, increased from the baseline radiograph obtained on day 1 (not shown). **B** Axial CT (lung window) obtained postoperative day 5 after slow weaning from mechanical ventilation, shows interlobular septal thickening (green arrowhead), groundglass opacity (green arrow), bibasilar atelectasis (blue arrows), and pleural effusions (yellow arrowheads), representing typical HRCT findings of pulmonary edema. Diagnosis of grade 3 PGD was made based on a PaO2/FiO2 ratio of < 200 and the radiologic findings

Presentation and imaging features are similar to ARDS [30], including mid to lower lung perihilar airspace opacities on radiograph and diffuse consolidative or mixed consolidative and ground glass opacities, interlobular and peribronchial interstitial thickening on CT (Fig. 7) [2]. PGD has a significant impact on both early and late prognoses, including its association with later development of BOS. PGD is a clinical diagnosis and thus transbronchial biopsies are not routinely performed [2, 11].

### Pleural

Pleural effusion and pneumothorax are common in the immediate postoperative period. Persistent air leak beyond one week may lead to persistent/increasing pneumothorax, pneumomediastinum, and subcutaneous emphysema on subsequent radiographs, which would require investigation for a causative airway complication [14, 31]. Persistent/increased effusions should suggest pathology such as hemothorax, chylothorax, and empyema [58]. Clinical presentations of pleural complications vary based on etiology. Any large volume effusion will produce mass effect and patients may experience non-specific symptoms such as dyspnea and cough [59].

**Fig. 8:**
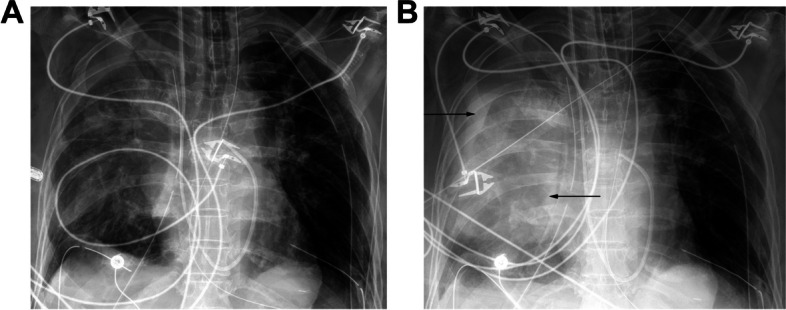
60-year-old female who developed hemothorax immediately after single lung transplant. **A** Frontal chest radiograph obtained immediately after surgery shows right sided lung transplant with support devices, a layering pleural effusion overlying the lung apex, and hazy airspace opacities. **B** Frontal chest radiograph obtained later the same day shows rapid opacification of the right lung transplant with contralateral mediastinal shift due to a large layering effusion (arrows). Findings were suspicious for rapidly developing hemothorax. The patient was taken to surgery, confirming hematoma in the pleural space, with subsequent evacuation and resolution (not shown)

Hemothorax usually occurs in the early post-operative period. CT will demonstrate hyperdense pleural fluid of 35–70 Hounsfield units. Rapid pleural fluid accumulation on early radiographs is another sign of hemothorax (Fig. 8) and would likely require an emergent return to the operating room if in the early postoperative period [2, 15].

**Fig. 9:**
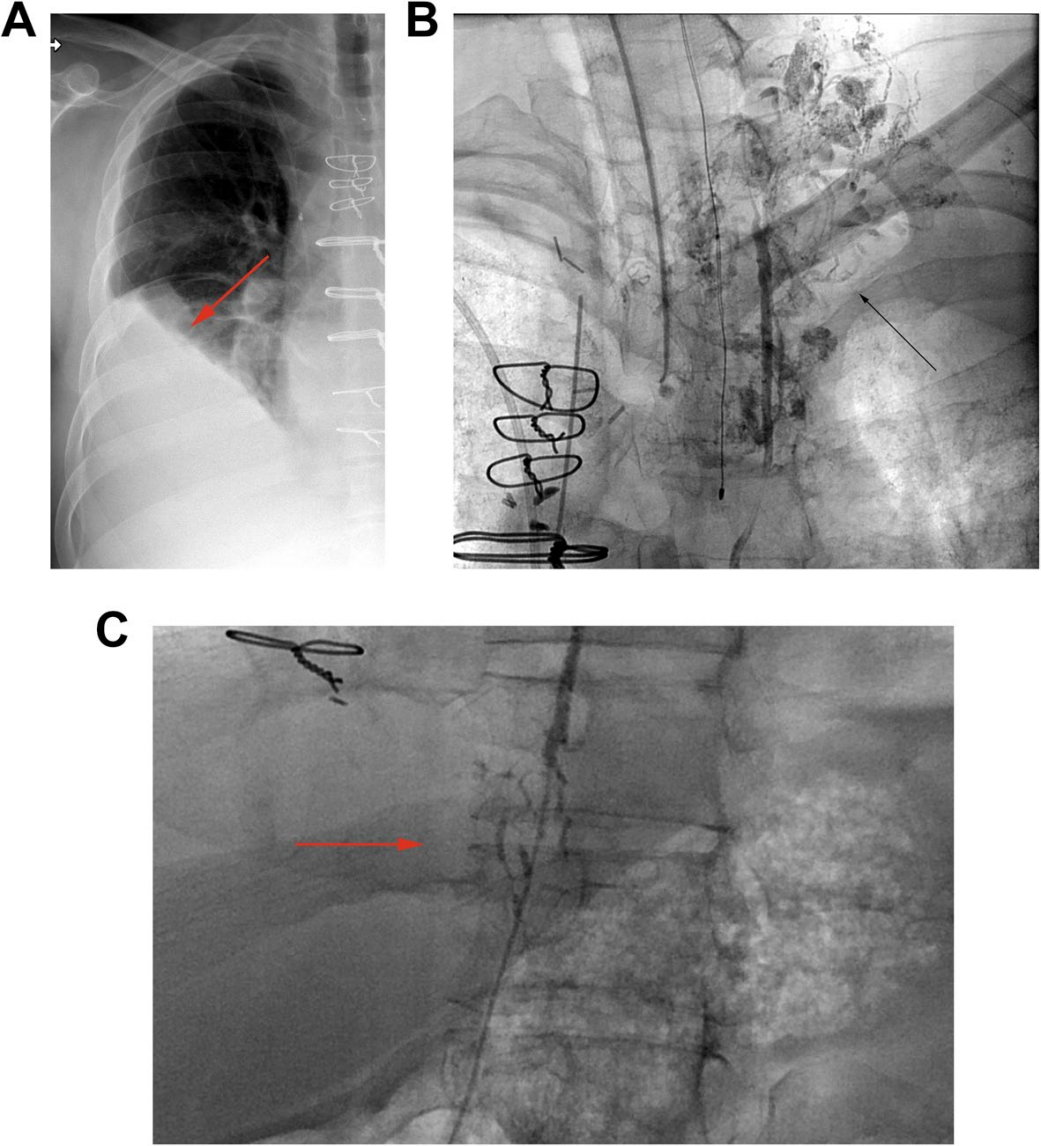
36-year-old male who underwent bilateral lung transplants for cystic fibrosis who developed chylothorax 3 weeks after surgery. **A** Frontal chest radiograph obtained for worsened dyspnea and hypoxia shows a moderate sized (arrow), increased from previous exams (not shown) and greater than expected for the postoperative time frame. Pleural fluid analysis demonstrated milky fluid with elevated white blood cell and triglyceride levels consistent with chylothorax. **B, C** Fluoroscopic angiographic images from lymphangiography performed by the interventional radiology service due to persistent chylous effusion despite surgical thoracic duct ligation and talc pleurodesis. There is extravasation of contrast at the level of the mid thoracic duct (arrow in **B**) and at the level of the diaphragm (arrow in **C**) indicating chyle leak. The leak resolved after thoracic duct embolization

Chylothorax presents as a persistent or increasing effusion despite adequate chest tube drainage, though cannot be specifically diagnosed based on imaging features and requires pleural fluid analysis [15]. Elevated pleural triglycerides, chylomicrons, and lymphocytes are diagnostic (Fig. 9) [15]. Incidence from small cohort studies ranged from < 1–11%. Extensive pleural adhesions from prior infection or pleurodesis can increase risk of thoracic duct injury. Chylothorax is prevalent among patients with lymphangioleiomyomatosis (LAM) [60].

As infectious complications are common in this population, empyema should be considered and ruled out in the setting of a new pleural effusion, especially with loculation [2, 15]. While uncommon, it has been to shown to increase mortality [61, 62]. On CT, it appears as a loculated or lenticular shaped effusion with pleural thickening and enhancement resulting in the split pleura sign [15].

### Infection

Early infections are most commonly bacterial, and infection is the most significant factor of morbidity and mortality within the first year, although it remains an important complication to consider even afterwards [10, 16, 63]. In addition, infections and colonization with organisms such as *Pseudomonas, Cytomegalovirus* (CMV), and *Aspergillus*, have been linked to higher rates of CLAD and acute rejection [64]. Bronchoscopy with bronchoalveolar lavage (BAL) and transbronchial biopsies are useful adjuncts to the diagnosis of bacterial and opportunistic infections [63, 65]. Tracheobronchial infections are often found incidentally on surveillance bronchoscopies, however if narrowing of the airway occurs due to fibrinous debris or mucus, patients may present with signs of airway obstruction such as a drop in FEV1, dyspnea, and/or wheezing [29, 64].

Bacterial infection is the leading cause of mortality up to 6 months [10]. Risk factors for infection include immunosuppression, prolonged mechanical ventilation, and bronchial mucosal ischemia [66]. While radiograph is often first line imaging, CT will reveal consolidations, pleural effusions, centrilobular nodules, tree-in-bud opacities, and interlobular septal thickening with greater sensitivity [16, 63].

**Fig. 10:**
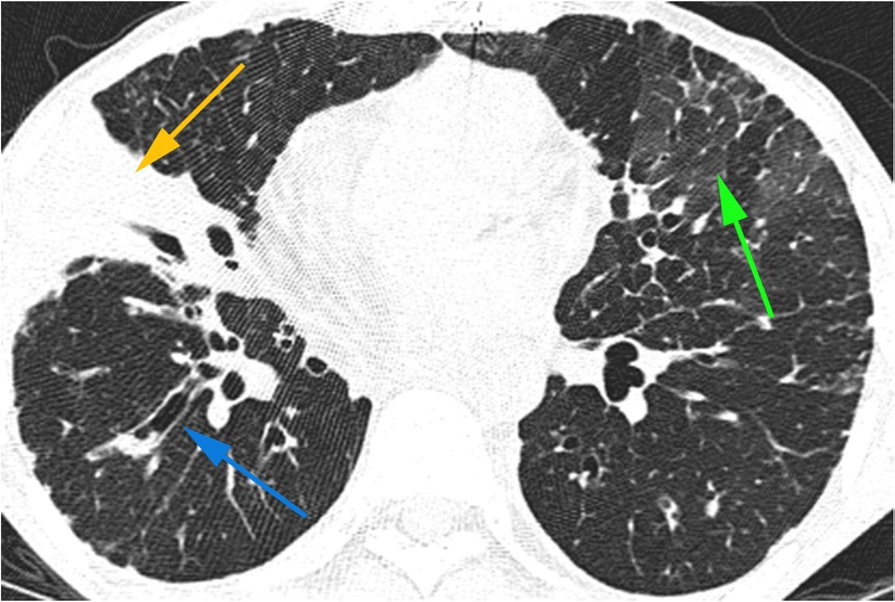
BCC in a 38-year-old male 3 years out from bilateral lung transplants for cystic fibrosis. Axial CT image (lung window) at the level of the lower lobes shows right middle lobe consolidation and atelectasis (yellow arrow) with bronchiectasis (blue arrow) that had increased from postoperative baseline, as well as scattered ground glass opacities with interlobular septal thickening (green arrow). The patient was admitted for chest pain, dyspnea, and hypoxia and was found to have *Pseudomonas* and *Burkholderia cepacia* on sputum culture

*Burkholderia cepacia* complex (BCC) affects 3–6% of CF patients [67]. The specific genomovar III, *B cenocepacia,* has the highest incidence of “cepacia syndrome,” which presents with necrotizing pneumonia and sepsis [67, 68]. Up to 80% mortality at 1 year has been documented in retrospective studies [67]. CF patients are the most likely to be colonized with BCC, and can undergo a rapid decline in lung function as well as cepacia syndrome. Most transplant centers consider BCC colonization an absolute contraindication to transplant in CF patients due to decreased survivability [67–69]. CT showing mucoid impaction suggests BCC colonization, while rapid progression of bronchiectasis and consolidations correlates with either graft function decline or cepacia syndrome (Fig. 10) [70–72].

Viral and fungal infections occur late after transplant. The most common viral pneumonia is due to CMV, which takes place in the setting of seronegative mismatch (seronegative recipient and seropositive donor) and/or non-adherence to prophylaxis [2, 63]. Widespread use of prophylaxis as well as quantitative polymerase chain reaction (PCR) assays monitoring for viremia has resulted in decreased incidence [63]. In immunosuppressed transplant patients, clinical presentation can range from non-specific features such as myalgia, arthralgia, and leukopenia to multi-organ system involvement [63]. Imaging findings include ground glass opacities or consolidations, as well as diffuse nodular pattern with tree-in-bud opacities [2, 16, 73].

**Fig. 11:**
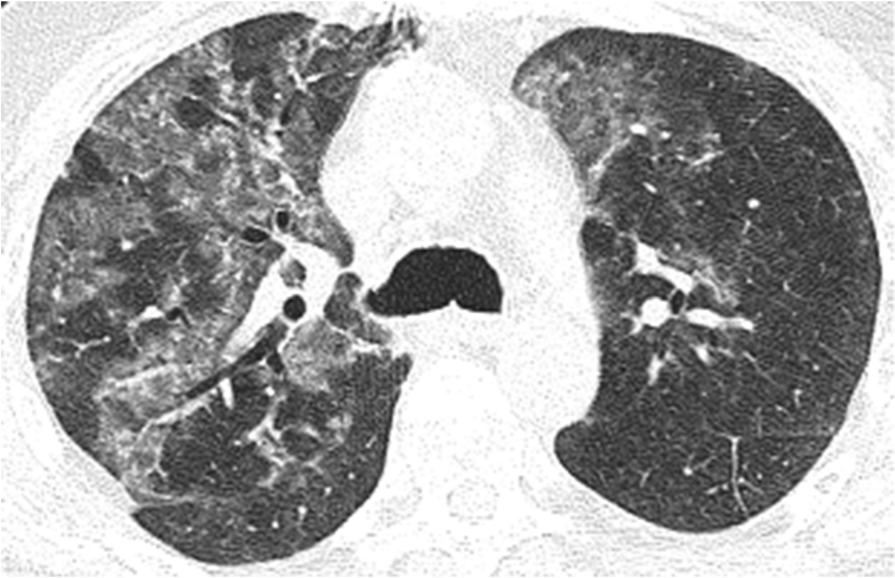
PJP/PCP in a 59-year-old male 3 years out from bilateral lung transplants for idiopathic pulmonary fibrosis. Axial CT image (lung window) at the level of the upper lobes shows right greater than left peribronchovascular distribution of ground glass opacities, which increased on subsequent imaging during that admission (not shown). The patient was originally admitted with fever and constitutional symptoms with suspicion for post-transplant lymphoproliferative disorder, however diagnosis of PJP/PCP was consistent based on the imaging, history of non-adherence to prophylaxis for approximately one year, and sputum PCR results

Typical CT features of fungal infections are nodular ground glass or consolidative opacities, including the “halo” sign of consolidations with peripheral ground glass [2]. *Pneumocystis jirovecii* pneumonia (PJP/PCP), like CMV, is more common with non-adherence to prophylaxis [14]. Patients typically present with hypoxemia out of proportion to other clinical findings [63]. Typical imaging findings are bilateral subpleural sparing ground glass opacities (Fig. 11), with interlobular septal thickening, nodular opacities, and apical predominant cystic lesions that can result in pneumothorax and pneumomediastinum [14].

Late-presenting infections include tuberculosis (TB) and non-tuberculous mycobacterial infection (NTM), with lung transplant recipients at higher risk compared to other solid organ recipients [64]. CT of mycobacterial infection shows centrilobular or tree in bud nodules, miliary nodular pattern, consolidations, bronchiectasis, and cavitations [10]. NTM, commonly including *Mycobacterium abscessus*, results in increased mortality [63, 64]. The diagnosis of NTM infection requires specific radiologic findings and positive sputum culture, BAL, or transbronchial biopsy [63, 74].

**Fig. 12:**
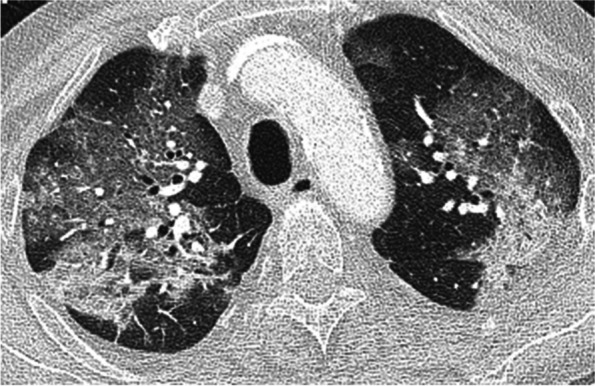
Severe Covid-19 pneumonia in a 69-year-old male with a history of lung transplants 10 years ago. Axial CT image (lung window) shows extensive bilateral patchy as well as confluent ground glass opacities. Covid-19 infection was confirmed with nasopharyngeal swab. The patient required mechanical ventilation

Retrospective studies report severe presentation and higher mortality rates in transplant recipients with COVID-19 compared to the general population [64, 75]. Radiologic findings are similar whether transplanted lungs are present or not (Fig. 12). Confounding features are possible in patients with SLTs with native lung fibrosis or emphysema [76]. Recent studies with at least 6 month follow up of these patients demonstrated allograft function decline following severe COVID-19 infection [77, 78]. New cases of CLAD including BOS and restrictive allograft syndrome (RAS) phenotypes have been reported, with prior studies demonstrating a link between viral pneumonia and CLAD [79, 80]. Long term follow-up should establish whether allograft dysfunction and imaging findings are permanent [76, 77].

### Immunologic

Hyperacute rejection is primarily described in case reports, as most cases are averted through anti-HLA antibody screening and avoidance of organs bearing those specific HLA antigens [30]. It presents intraoperatively or within the first few hours after the vascular anastomosis is created [10], due to rejection by antibodies reactive to the allograft already in the recipient’s circulation [81]. It can present as severe and refractory hypoxemia [2], with rapid airspace opacification on imaging [10].

Acute cellular rejection (ACR) most commonly occurs in the early period, radiographically evident by 5–10 days, though still possible weeks to months after transplant [2, 10]. The pathophysiology involves a T-cell mediated immune response by the recipient against donor allograft antigens [2, 15]. 27.3% of transplant recipients had a previously treated episode of acute rejection at one year in the 2019 ISHLT report. 2.7 and 1.9% of all transplant patient deaths at 30 days and 1 year respectively were attributed to acute rejection [6, 82].

Presentation is similar to infection, including dyspnea, fever, and leukocytosis [30]. At imaging, CT shows interlobular septal thickening, centrilobular ground glass opacities, and scattered to diffuse areas of ground glass and consolidative opacities [2, 15]. Despite this, CT has limited detection of acute rejection and as such tissue sampling via transbronchial biopsy is required for diagnosis [2, 83]. There is a strong association with the development of BOS [84].

The 2016 ISHLT consensus report on antibody-mediated rejection (AMR) provided standardized diagnostic criteria, including allograft dysfunction, the presence of donor specific antigens (DSA), and suggestion of AMR on histology including positive staining for complement component 4d (C4d) [81]. While associated with hyperacute and acute forms of rejection, AMR also appears weeks to months out from transplantation. Antibodies specific to the allograft may be pre-formed prior to actual transplantation particularly in the setting of hyperacute rejection, while AMR outside the hyperacute setting is typically the result of sensitization to DSAs after transplantation [84]. A recent multicenter prospective study found an incidence of 47% among 335 lung transplants, including clinical and subclinical AMR. Significantly increased risk of CLAD and death was also found [85].

Imaging findings of AMR are nonspecific, with ground glass opacities and air trapping on expiratory CT reported in certain studies. Imaging is more useful in the exclusion of other etiologies such as infection [85, 86]. Transbronchial biopsy may be helpful, although the appearance of certain histological features such as pulmonary capillary injury may be non-specific [87, 88]. AMR and DSA positivity are associated with CLAD [81, 87].

**Fig. 13:**
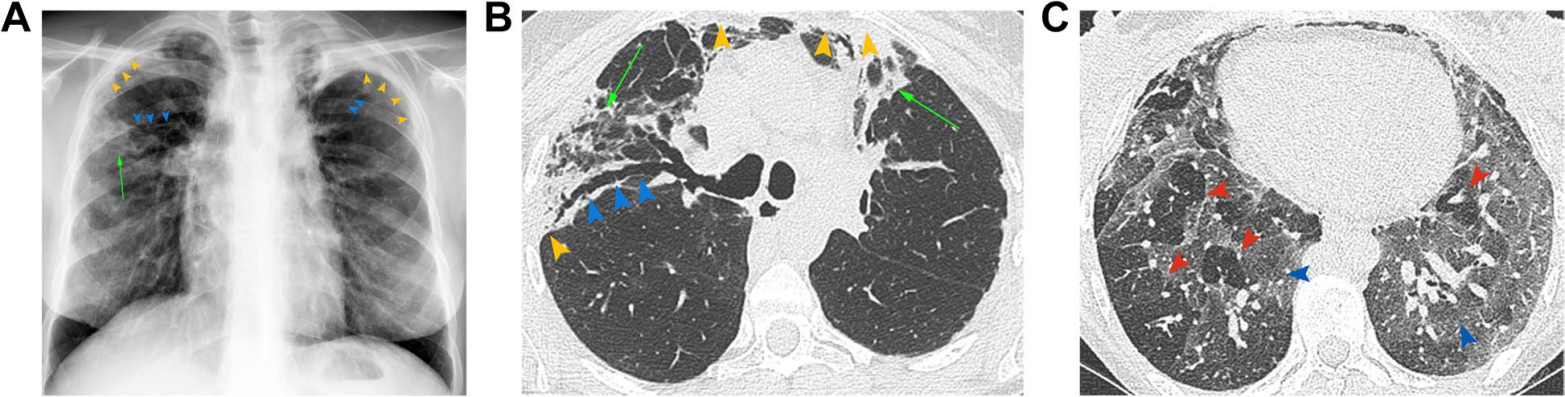
CLAD with mixed BOS/RAS features in a 37-year-old female 13 years out from lung transplants for cystic fibrosis. **A** Frontal chest radiograph 9 months after Covid-19 infection in this patient with persistent dyspnea and ventilatory defect on PFTs shows apical predominant pleural thickening (yellow arrowheads), bronchiectasis (blue arrowheads) and confluent fibrosis (green arrow). **B** Axial CT image (lung window) at the upper lobes (**B**) again shows areas of pleural thickening (yellow arrowheads) with subpleural confluent fibrosis (green arrow) and traction bronchiectasis (blue arrowheads). Findings are typical of pleuroparenchymal fibroelastosis. **C** Axial static end-expiratory CT image at the level of the lower lobes (lung window) shows lobular air trapping (red arrowheads) with intervening dense normal lung (blue arrowheads), representative of small airways disease and indicative of BOS

CLAD is defined as persistent ≥ 20% decline in FEV_1_ from baseline, through obstructive, restrictive, mixed, or undefined patterns on pulmonary function tests (PFTs) [89]. The most common presenting phenotypes are BOS and RAS (Fig. 13) [2, 89]. The incidence of CLAD is 50% at 5 years and 76% at 10 years [82]. Risk factors include PGD, ACR, AMR, gastroesophageal reflux, infection, and non-adherence to immunosuppressive medications [30]. Glucagon-like peptide-1 receptor agonists, used to treat solid organ recipients with diabetes mellitus, can cause gastroparesis and intensify gastrointestinal complications commonly seen in patients on certain immunosuppressants, theoretically leading to poor tolerance [90].

BOS is characterized by an obstructive (FEV1/FVC < 0.7) spirometry pattern without restriction [89]. BOS comprises 65–75% of cases of CLAD with an incidence of 8.8% at 1 year and 41.1% at 5 years, with median survival of 3–5 years [89, 91]. CT shows expiratory air trapping, bronchial wall thickening, and bronchiectasis (Fig. 13). Other than to exclude other causes of decline in spirometry, bronchoscopy and histopathology have limited utility in diagnosis [2, 30, 91]. The use of quantitative imaging in combination with a radiologist’s qualitative analysis has also been shown to increase diagnostic performance in distinguishing BOS from other entities [92].

Diagnostic criteria for RAS include a restrictive pattern in spirometry (persistent ≥ 10% decline of TLC from baseline), absence of airflow obstruction, and persistent opacities on imaging [89]. RAS comprises 25–35% of CLAD cases with a median survival of 6–18 months [91]. The incidence of RAS and mixed phenotypes is 14–26% [93]. Both PFT and radiologic abnormalities persist despite treatment [15, 30, 91]. Histopathology reveals either acute inflammation or pleuroparenchymal fibroelastosis (PPFE). Radiologic features of PPFE include apical predominant pleural thickening with subjacent reticular opacities, traction bronchiectasis, and architectural distortion (Fig. 13) [94, 95].

### Primary recurrence

Recurrence of primary disease may occur months to years after surgery [30]. A multicenter retrospective study [96] showed 1% recurrence in 1,354 transplant recipients. Sarcoidosis was the most common to recur, in approximately 33% of cases. Imaging of recurrent sarcoid shows solitary, perilymphatic, or miliary nodules, and lymphadenopathy [96]. Other reported examples of recurrence include LAM and pulmonary alveolar proteinosis [14, 30].

**Fig. 14:**
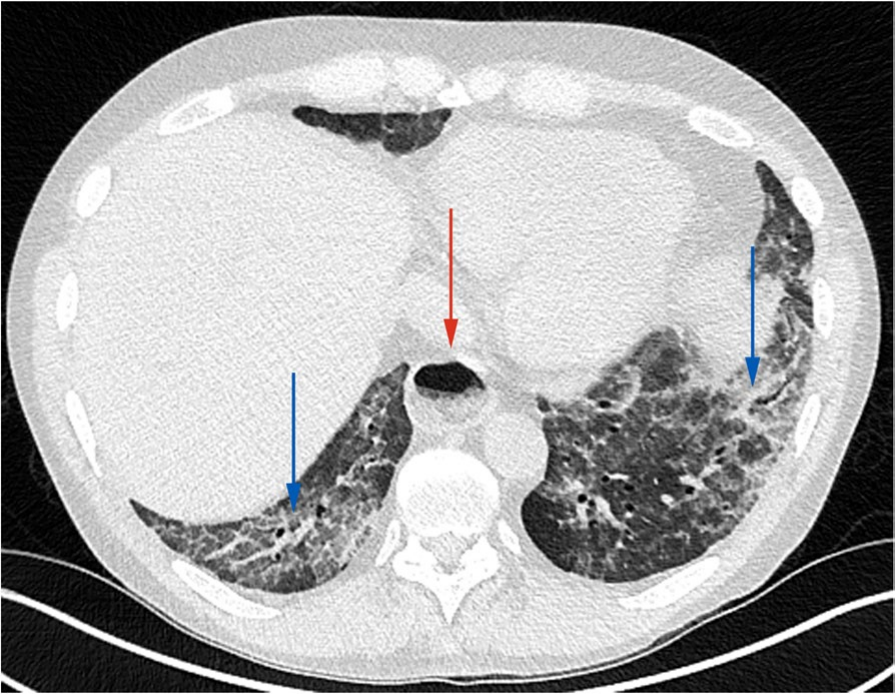
Recurrent CTD-ILD (NSIP pattern) in a 48-year-old male approximately 1 year out from lung transplant for scleroderma ILD and pulmonary hypertension. Axial CT (lung window) at the level of the lung bases shows bases shows ground glass density and traction bronchiolectasis with areas of subpleural sparing (blue arrows). A patulous debris filled esophagus is present (red arrow), consistent with esophageal dysmotility. In the setting of opacities, rising troponin and creatine kinase, and new gastroesophageal reflux, BAL with biopsy was performed which was negative for rejection and showed elevated eosinophils. The presumptive diagnosis of scleroderma-ILD recurrence was made

While interstitial lung disease (ILD) makes up approximately 40% of transplant indications [3, 6], recurrence is infrequently reported in the literature, especially connective tissue disease (CTD) associated ILD (CTD-ILD) (Fig. 14), hypersensitivity pneumonitis, and desquamative interstitial pneumonia. Risk factors are unknown [52]. A high index of suspicion is necessary in transplant recipients with either a history of or suspected CTD/CTD-ILD [97].

### Malignancy

**Fig. 15:**
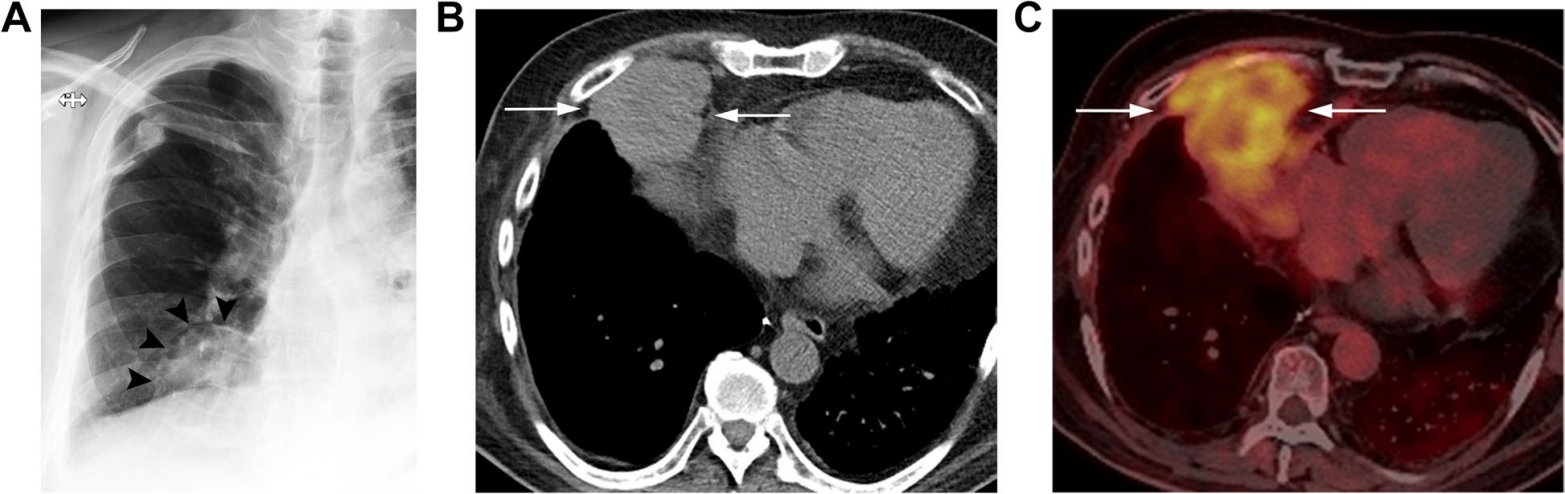
PTLD in a 72-year-old male 1 year out from single lung transplant. **A** Frontal radiograph shows a mass in the medial right lower lung (arrowheads). **B** Axial unenhanced CT (mediastinum window) shows a circumscribed soft tissue density mass (arrows) in the right cardiophrenic angle. **C** Axial PET-CT shows heterogeneously increased FDG uptake in the mass (arrows). No other FDG-avid lesions were identified. Tissue sampling showed monomorphic PTLD, which was EBV positive

Posttransplant lymphoproliferative disorder (PTLD) represents a heterogeneous group of lymphoid disorders, and involves the allografts as early as 1 month, though typically within the first year after transplant, with a greater incidence in Epstein-Barr virus (EBV) seronegative and immunocompromised populations [30, 98]. Incidence is between 3–9% [98], with mortality at 30–75% [99]. The most common intrathoracic radiologic manifestations of PTLD include homogenous solitary intraparenchymal mass or solitary or multiple nodules, less commonly intrathoracic lymphadenopathy [30]. Findings are hypermetabolic on positron emission tomography(PET)-CT [30] (Fig. 15) which increases sensitivity and staging accuracy [99]. The imaging features in combination with the timing of clinical presentation, lab abnormalities, and allograft dysfunction should direct toward the need for tissue sampling [100].

Immunosuppression contributes to increased risk of development and progression of primary lung neoplasm [30]. Lung cancer rates are up to 5.5 times higher than that of the general population, with incidence of 1–9% [2, 98]. Transplant recipients have higher morbidity and mortality [98]. Pulmonary malignancy can be found de novo, in the native lung from SLTs, incidentally from lung explants, or recurrent. Donor malignancy is rare [98].

## Conclusion

While the numbers of transplants performed have been increasing, improvement in survival still lags behind other solid organ transplant recipients. Radiology can play a crucial role in screening for and detecting transplant complications, a significant cause of recipient morbidity and mortality. Therefore, it is crucial for both radiologists and transplant clinicians to have a broad awareness of the timeline and diagnostic findings of possible complications.

### Supplementary Information

**Supplementary Material 1.**

**Supplementary Material 2.**

## Data Availability

No datasets were generated or analysed during the current study.

## Abbreviations

A1ATD: Alpha-1 antitrypsin deficiency
ACR: Acute cellular rejection
AMR: Antibody mediated rejection
ARAD: Azithromycin responsive allograft dysfunction
ARDS: Acute respiratory distress syndrome
BAL: Bronchoalveolar lavage
BCC: Burkholderia cepacia complex
BLT: Bilateral lung transplants
BOS: Bronchiolitis obliterans syndrome
C4d: Complement component 4d
CF: Cystic fibrosis
CLAD: Chronic lung allograft dysfunction
CMV: Cytomegalovirus
COPD: Chronic obstructive pulmonary disease
CT: Computed tomography
CTA: Computed tomography angiography
CTD: Connective tissue disease
DSA: Donor specific antigen
EBV: Epstein Barr virus
FEV1: Forced expiratory volume
FiO2: Fraction of inspired oxygen
FVC: Forced vital capacity
HLA: Human leukocyte antigen
ILD: Interstitial lung disease
ISHLT: International society for heart and lung transplantation
LAM: Lymphangioleiomyomatosis
MR: Magnetic resonance
mTOR: Mechanistic target-of-rapamycin
NTM: Non-tuberculous mycobacterium
PA: Pulmonary artery
PAH: Pulmonary arterial hypertension
PaO2: Partial pressure of oxygen
PAP: Pulmonary artery pressure
PCR: Polymerase chain reaction
PE: Pulmonary embolism
PET: Positron emission tomography
PGD: Primary graft dysfunction
PJP/PCP: Pneumocystis jirovecii pneumonia/pneumocystis pneumonia
PPFE: Pleuroparenchymal fibroelastosis
PTLD: Post-transplant lymphoproliferative disorder
RAS: Restrictive allograft syndrome
RV: Right ventricle
SLT: Single lung transplant
TB: Tuberculosis
TBM: Tracheobronchomalacia
TLC: Total lung capacity
VBIS: Vanishing bronchus intermedius syndrome
VTE: Venous thromboembolism
