# Supplementary material for: Pulmonary transplant complications: a radiologic review
Source: J Cardiothorac Surg. 2024 May 3;19:270. doi: 10.1186/s13019-024-02731-w (PMC11067284; doi:10.1186/s13019-024-02731-w)
Supplement: Supplementary file 1 — Supplementary Material 1. [file 13019_2024_2731_MOESM1_ESM.docx]

| **Scanning instructions** |  |
| --- | --- |
| Use Flash mode if available. |  |
| Position | Supine |
| Topogram | AP |
| Contrast | None |
| Coverage* | Lung apices through adrenal glands |

Two exams done, end inspiration and dynamic expiration. See below for details.

**Inspiration**: Technique: Low dose 120 kVp, 40 effective mAs, spiral mode on end inspiration 1mm x 1mm B31f

|  | **Recon Type** | **Slice/Increment (mm)** | **Algorithm** | **Window** | **Networking** | **Post-Processing** | **Field of view** |
| --- | --- | --- | --- | --- | --- | --- | --- |
| Recon 1 | Axial | 5 x 5 | B60f Sharp | Lung | PACS | None | Lung field |
| Recon 2 | Axial | 5 x 5 | B30f Medium Smooth | Soft tissue | PACS | None | Body Contour |
| Recon 3 | Axial (for coronal recons) | 3 x 1.5 | B30f Medium Smooth | Soft tissue | PACS | None | Body Contour |
| Recon 4 | Coronal | 5 x 5 | B30f Medium Smooth | Soft tissue | PACS | None | Body Contour |
| Recon 5 | Axial (3D-MIP Thin) | 8 x 3 | B35f or i36f kernel Medium Smooth | Lung | PACS | None | Lung Field |

**Expiration:** Technique: Low dose 120 kVp, 40 effective mAs, 2.5 mm collimation, pitch of 1.5. Spiral mode during **dynamic expiration****

*Coverage: Techs should check to see if the membranous portion of the trachea (the posterior wall) is bowed anteriorly to be certain the expiratory scan was obtained correctly. If it was missed, repeat the expiratory dataset.

**Patients should be coached with instructions to take a deep breath and to blow it out during the image acquisition, which must be coordinated to begin with the onset of the patient's forced expiratory effort. So, the patient will be exhaling during image acquisition.

**Supplemental Table 1.** CT Protocol for Tracheobronchomalacia

Note.--- kVp = kilovoltage peak, mAs = milliampere-seconds, MIP = maximum intensity projection, PACS = Picture Archiving and Communication System
