# Supplementary material for: Pulmonary transplant complications: a radiologic review
Source: J Cardiothorac Surg. 2024 May 3;19:270. doi: 10.1186/s13019-024-02731-w (PMC11067284; doi:10.1186/s13019-024-02731-w)
Supplement: Supplementary file 2 — Supplementary Material 2. [file 13019_2024_2731_MOESM2_ESM.docx]

| **Scanning instructions** |  |
| --- | --- |
| Position | Supine |
| Topogram | AP |
| Contrast | None |
| Coverage | Chin through lung bases |
| Care Dose/Care kV* | Use if scanner allows (quality reference mAs 140, reference kVp 100) |

Scan using maximum inspiration breath hold.

Send last two recons to 3DR for trachea post processing.

|  | **Recon Type** | **Slice/Increment (mm)** | **Algorithm** | **Window** | **Networking** | **Post-Processing** | **Field of View** |
| --- | --- | --- | --- | --- | --- | --- | --- |
| Recon 1 | Axial | 5 x 5 | I31f Medium smooth | Mediastinum | PACS | None | Body Contour |
| Recon 2 | Axial | 5 x 5 | I70f Very sharp | Lung | PACS | None | Lung Field |
| Recon 3 | Coronal | 3 x 3 | I31f Medium smooth | Mediastinum | PACS | None | Body Contour |
| Recon 4 | Sagittal | 3 x 3 | I31f Medium smooth | Mediastinum | PACS | None | Body Contour |
| Recon 5 | Axial (3D-MIP Thin) | 8 x 3 | I31f Medium smooth | Lung | PACS | None | Lung Field |
| Recon 6 | Axial | 1 x 1 | I70f Very sharp | Lung | PACS 3DR | 3DR | Full scan range |
| Recon 7 | Axial | 1 x 1 | I31f Medium smooth | Mediastinum | PACS 3DR | 3DR | Full scan range |

**Supplemental Table 2**. CT Protocol for 3D Tracheobronchial Reconstruction

Note.--- 3DR = 3D reconstruction, kVp = kilovoltage peak, mAs = milliampere-seconds, MIP = maximum intensity projection, PACS = Picture Archiving and Communication System
